# Carcinoid Heart Disease Presenting as Cardiogenic Shock: A Case Report

**DOI:** 10.7759/cureus.115309

**Published:** 2026-08-27

**Authors:** Pedro Duque, Yisseth Salazar-Ramírez, Alexandra Guzman, Andres Arango, Samir Alexander Pantoja

**Affiliations:** 1 Faculty of Medicine, Universidad de Antioquia, Medellin, COL; 2 Medicine, Industrial University of Santander, Bucaramanga, COL; 3 Critical Care, Clínica Las Américas, Medellin, COL; 4 Internal Medicine, Universidad de Antioquia, Medellin, COL

**Keywords:** carcinoid heart disease, carcinoid tumor, cardiogenic shock, malignant carcinoid syndrome, right heart failure

## Abstract

Carcinoid heart disease (CHD) is a complication of carcinoid syndrome that results from chronic exposure of the endocardium to circulating vasoactive substances, particularly serotonin. It leads to progressive right-sided valvular dysfunction and right heart failure. Presentation as cardiogenic shock is uncommon and may delay recognition of the underlying disease.

We present the case of a 44-year-old woman with no prior medical history who developed progressive dyspnea, ascites, and lower extremity edema over four months and was admitted with cardiogenic shock. Transthoracic echocardiography revealed massive tricuspid regurgitation and moderate-to severe pulmonary regurgitation with marked right ventricular dilation with preserved conventional systolic indices. Abdominal imaging demonstrated hepatomegaly with multiple hypervascular hepatic lesions, and liver biopsy confirmed metastatic neuroendocrine tumor. Urinary 5-hydroxyindoleacetic acid was markedly elevated at 305 mg/24 hours with pro-B-type natriuretic peptide (pro-BNP) at 2,450 pg/mL, supporting the diagnosis of carcinoid syndrome with cardiac involvement. Lanreotide was initiated for biochemical and symptomatic control of the underlying carcinoid syndrome. Despite intensive hemodynamic support with inotropic and vasopressor therapy, the patient developed refractory cardiogenic shock and died from progressive right-sided heart failure. This case highlights an unusual and aggressive presentation of carcinoid disease manifesting as cardiogenic shock. Clinicians should maintain a high index of suspicion for CHD in patients with unexplained right-sided valvular dysfunction and systemic congestion, as early recognition may allow timely therapeutic intervention.

## Introduction

Carcinoid syndrome is a well-recognized complication of metastatic neuroendocrine tumors and is strongly associated with cardiovascular involvement. It occurs as a result of the systemic release of vasoactive substances that escape hepatic metabolism and enter the systemic circulation, primarily serotonin. Persistent exposure to these mediators is linked to the development of carcinoid heart disease (CHD), which historically affected up to 50% of patients with carcinoid syndrome but is now reported in approximately 20% with contemporary treatment [1].

CHD is characterized by fibrotic plaque deposition rich in myofibroblasts at the endocardial surface, predominantly affecting right-sided valves. Progressive leaflet thickening, retraction, and loss of mobility lead to severe tricuspid and pulmonary regurgitation [1,2]. The hemodynamic consequence is chronic right ventricular volume overload, progressive dilation, and eventual right-sided heart failure.

Cardiac involvement significantly worsens prognosis. Three-year survival is approximately 31% in patients with CHD compared with 69% in those without cardiac disease. Once symptomatic heart failure develops, life expectancy in untreated patients may be limited to less than one year [2].

Diagnosis of CHD integrates the clinical context of carcinoid syndrome, biochemical markers, and characteristic valvular findings on cardiac imaging. Right-sided valvular involvement is present in approximately 90% of cases [3]. Among available biomarkers, N-terminal pro-B-type natriuretic peptide (NT-proBNP) has shown the best performance [4]. Advances in somatostatin analog therapy have improved symptom control and disease stabilization in patients with neuroendocrine tumors and carcinoid syndrome, yet mortality related to right-sided heart failure remains considerable [1].

We present the case of a 44-year-old woman whose initial manifestation of CHD was cardiogenic shock, illustrating how aggressive this condition can be in advanced cases and the importance of considering it in the differential diagnosis of unexplained right-sided heart failure.

## Case presentation

A 44-year-old woman with no prior medical history presented with a four-month history of progressive dyspnea, initially on moderate exertion and later with minimal activity, accompanied by orthopnea, ascites, and grade II-III lower-extremity edema. On admission, her blood pressure was 110/79 mmHg, heart rate was 84 beats/minute, respiratory rate was 16 breaths/minute, and oxygen saturation was 95% on room air. During echocardiographic assessment, she developed hypotension (85/57 mmHg) with a heart rate of 110 beats/minute. Clinical examination demonstrated delayed capillary refill, cold extremities, and persistent oliguria (0.2 mL/kg/hour), consistent with systemic hypoperfusion. Serum lactate was 2.76 mmol/L. Laboratory evaluation demonstrated acute kidney injury (creatinine, 2.4 mg/dL), while liver enzymes and bilirubin remained within normal limits (aspartate aminotransferase (AST), 30 U/L; alanine aminotransferase (ALT), 22 U/L; direct bilirubin, 0.48 mg/dL; indirect bilirubin, 0.29 mg/dL), although hypoalbuminemia (2.72 g/dL) was present. 

Initial management consisted of dobutamine (5 μg/kg/minute) and norepinephrine (0.05 μg/kg/minute), together with intravenous furosemide for volume overload. Due to persistent hypotension and an inadequate hemodynamic response, dobutamine was discontinued, and a 24-hour levosimendan infusion was initiated and titrated up to 0.2 μg/kg/minute while norepinephrine was continued.

**Figure 1:**
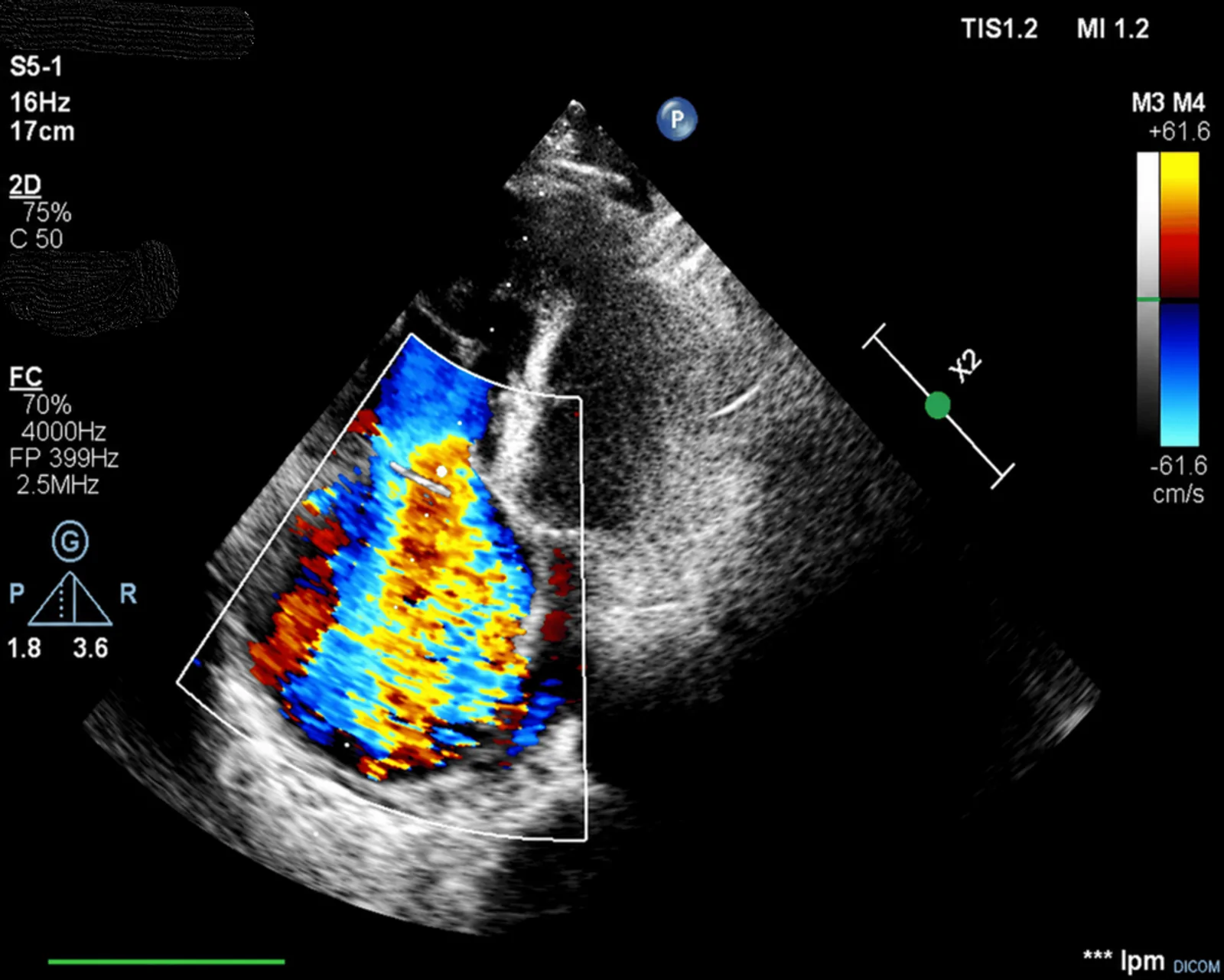
Transthoracic echocardiogram showing enlargement of the tricuspid annulus with tethered leaflets and a 16-mm coaptation gap, indicative of severe tricuspid regurgitation.

**Figure 2:**
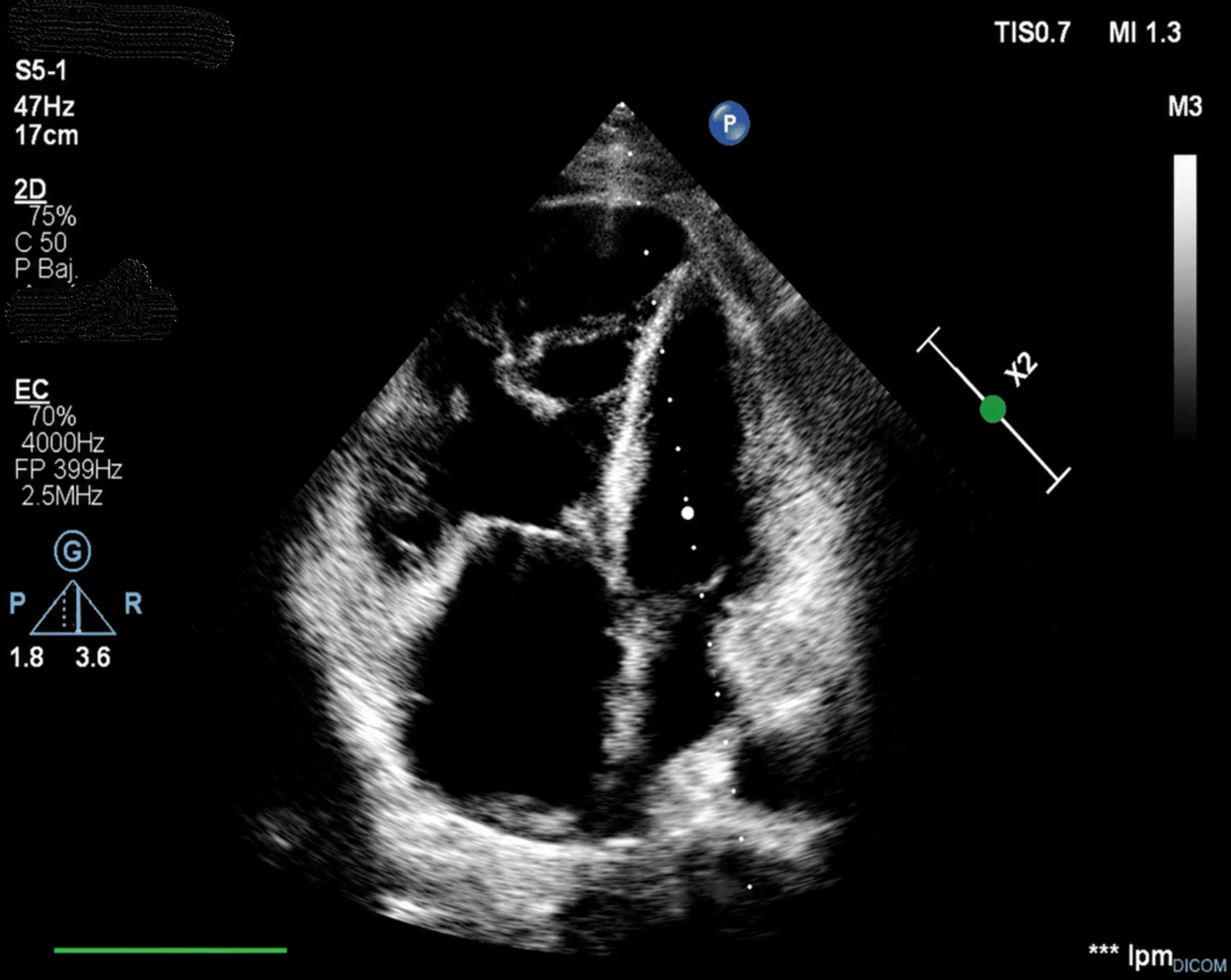
Transthoracic echocardiography demonstrating a severely dilated right ventricle (basal diameter, 54 mm; mid-cavity diameter, 41 mm. Quantitative right ventricular assessment showed a fractional area change (FAC) of 45%, free-wall longitudinal strain of -28%, tricuspid annular plane systolic excursion (TAPSE) of 17 mm, tissue Doppler S′ velocity of 12 cm/s, and an estimated pulmonary artery systolic pressure (PASP) of 42 mmHg.

Transthoracic echocardiography revealed massive tricuspid regurgitation and moderate-to-severe pulmonary regurgitation, accompanied by marked right ventricular dilation. Conventional indices of right ventricular systolic function (fractional area change (FAC), tricuspid annular plane systolic excursion (TAPSE), S′ velocity, and free-wall longitudinal strain) were borderline despite massive tricuspid regurgitation (Figures 1-2). 

Given the presence of isolated right-sided valvular disease and right ventricular failure, several alternative diagnoses were systematically considered. Pulmonary thromboembolism and chronic thromboembolic pulmonary disease were excluded by computed tomography pulmonary angiography and ventilation-perfusion scanning. Autoimmune connective tissue diseases were excluded through serologic testing. An infectious source was considered unlikely based on the absence of fever, negative blood cultures, and the lack of echocardiographic evidence of vegetations. Congenital tricuspid valve disease, rheumatic valvular disease, and drug-induced valvulopathy were excluded based on the characteristic echocardiographic findings of diffuse plaque-like thickening, leaflet retraction, and markedly reduced mobility involving both the tricuspid and pulmonary valves, findings highly suggestive of carcinoid heart disease.

**Figure 3:**
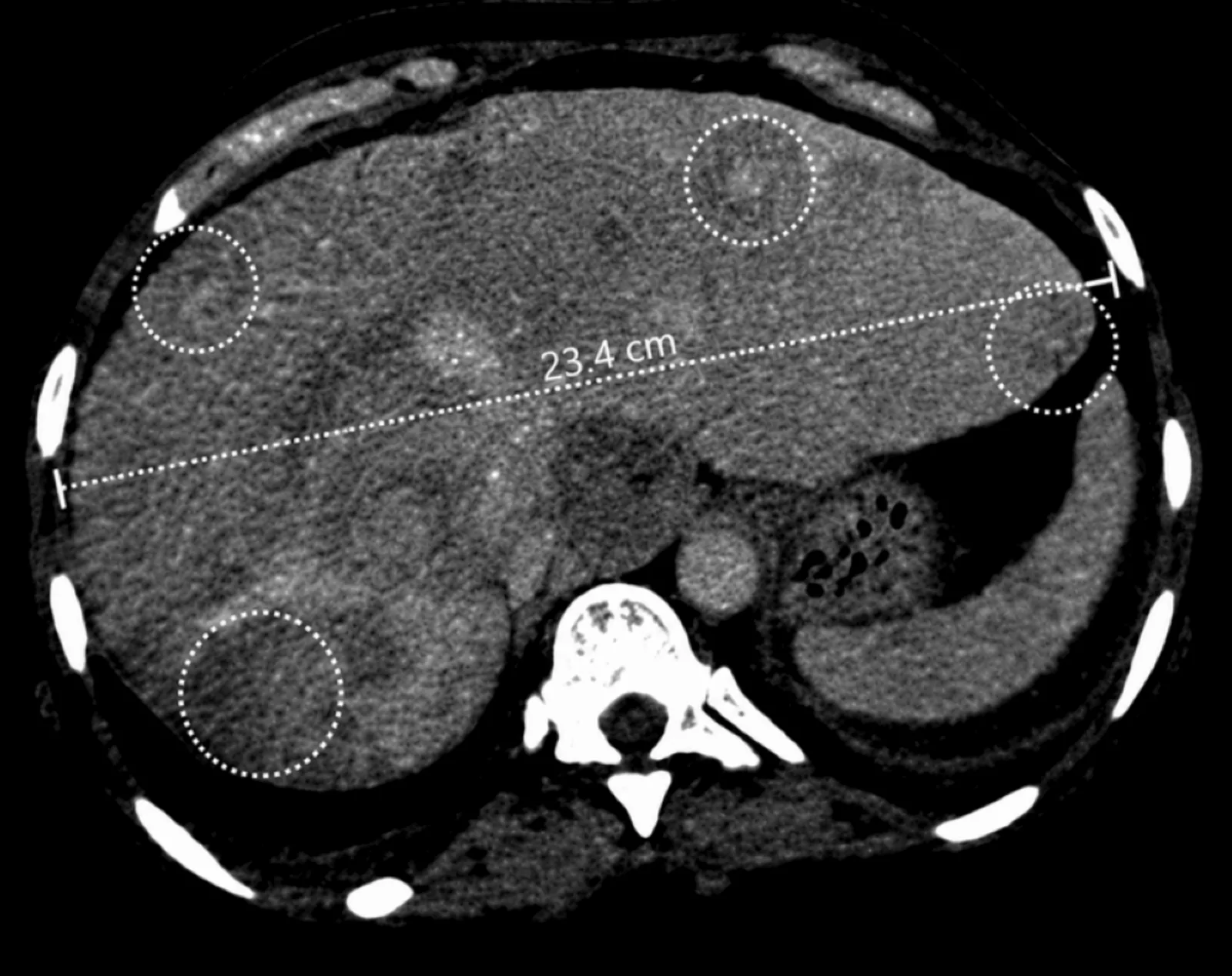
Contrast-enhanced axial CT of the abdomen demonstrating marked hepatomegaly (liver span, 23.4 cm). White dotted circles highlight multiple hypervascular hepatic lesions consistent with metastatic disease.

Abdominal ultrasound and contrast-enhanced computed tomography demonstrated marked hepatomegaly (23.4 cm) with multiple hypervascular hepatic lesions consistent with metastatic disease (Figure 3). Percutaneous liver biopsy revealed neoplastic cells arranged in trabecular and acinar patterns with uniform chromatin and low mitotic activity. Immunohistochemistry for synaptophysin and chromogranin was positive, confirming a neuroendocrine phenotype. The WHO grade and Ki-67 proliferation index were not available from the pathology report. Urinary 5-hydroxyindoleacetic acid (5-HIAA) was markedly elevated at 1,595 μmol/24 hour (305 mg/24 hour), and NT-proBNP was 2,450 pg/mL, supporting the diagnosis of carcinoid syndrome with carcinoid heart disease. Treatment with lanreotide 120 mg was initiated for biochemical and symptomatic control of carcinoid syndrome. Contrast-enhanced CT of the chest and abdomen did not identify the primary neuroendocrine tumor. Additional staging investigations, including functional imaging and endoscopic evaluation, could not be completed because of the patient’s critical clinical condition.

Despite intensive management in the Special Care Unit, the patient experienced a progressively deteriorating course marked by refractory cardiogenic shock and worsening renal function consistent with cardiorenal syndrome. She required prolonged inotropic and vasopressor support, with multiple unsuccessful attempts at weaning that required re-escalation of therapy, classifying her cardiogenic shock as Society for Cardiovascular Angiography and Interventions (SCAI) stage D.

The case was discussed by a multidisciplinary Heart Team. Surgical valve replacement, the only definitive therapy for established severe carcinoid valvular disease, was considered but deemed prohibitively high risk because of the patient's extreme clinical frailty and severe malnutrition, both of which were associated with a high risk of perioperative mortality. Transcatheter valve intervention was considered as a less invasive alternative but was not available at our institution.

**Figure 4:**
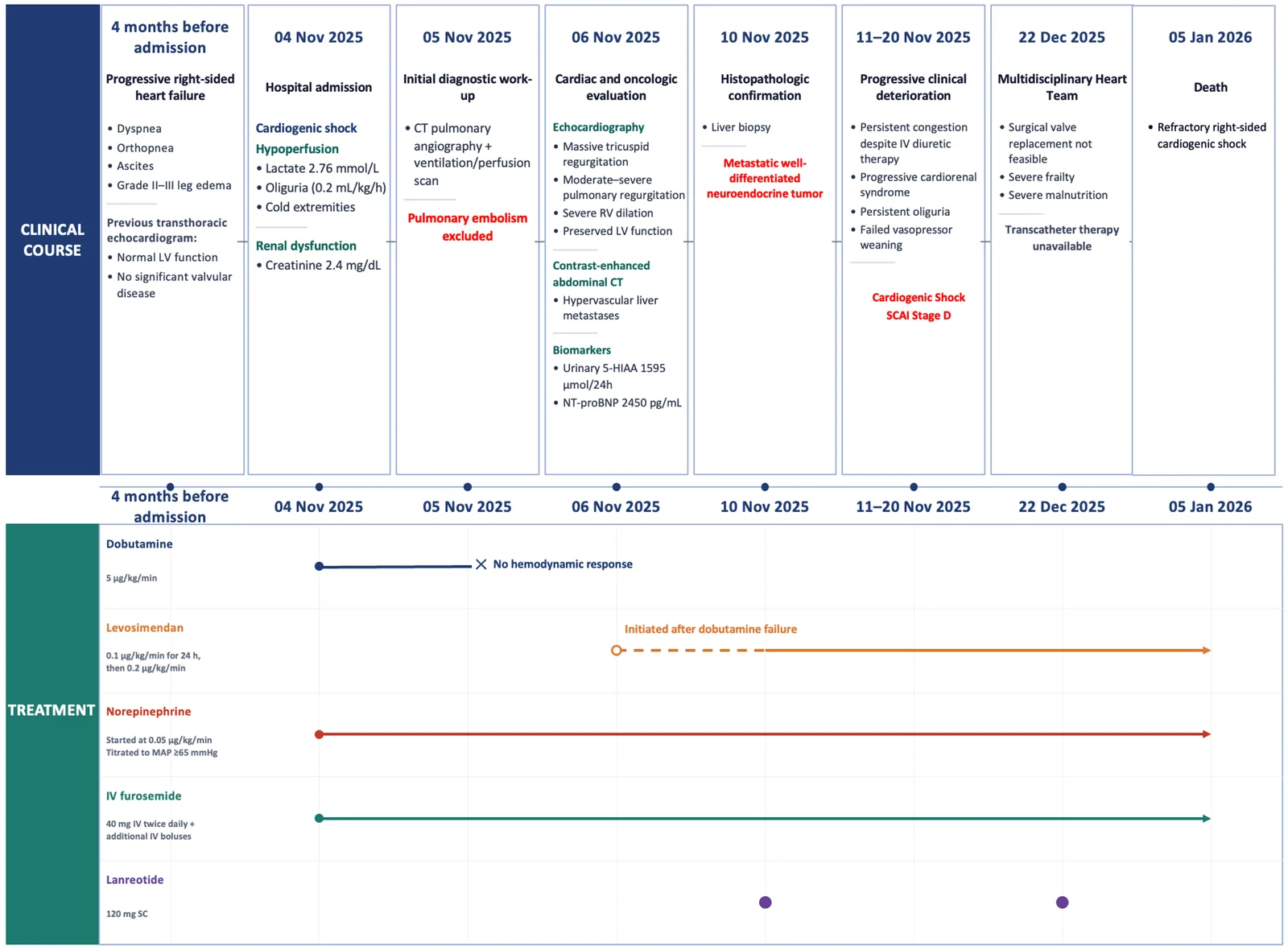
Timeline of clinical events. The image was created by the authors using Microsoft PowerPoint (Microsoft Corporation, Redmond, WA).

Despite intensive inotropic and vasoactive support, she died as a result of refractory cardiogenic shock caused by right-sided heart failure. The complete chronological sequence of the patient's clinical presentation and hospital course is summarized in Figure 4.

## Discussion

CHD is one of the most serious complications of carcinoid syndrome. Historically, it was reported in up to half of patients with carcinoid syndrome and liver metastases, but its prevalence has decreased to approximately 20% with contemporary treatment, including somatostatin analogues [1]. Most cases originate from tumors in the small intestine, with tumors in the lung, colon, or pancreas being less common [5]. 

CHD develops when vasoactive substances, particularly serotonin, kinins, prostaglandins, transforming growth factor-β, substance P, and chromogranin A circulate in the bloodstream and reach the right side of the heart, bypassing hepatic metabolism in the setting of liver metastases. These substances stimulate fibroblast proliferation and deposition of fibrous plaques on the valves and endocardium, leading to thickened, stiff, and leaky tricuspid and pulmonary valves [6]. Left-sided involvement is rare, as the lungs normally inactivate these mediators, which explains why right-sided heart failure predominates in >90% of patients [7]. Rare exceptions include cases associated with a patent foramen ovale or primary bronchopulmonary carcinoid tumors, in which these substances bypass pulmonary inactivation and reach the left heart.

An important aspect of this case is the diagnostic trajectory preceding hospital admission. Although the patient experienced progressive dyspnea, ascites, and peripheral edema over four months, these findings are nonspecific and may initially be attributed to more common causes of right-sided heart failure. Classical manifestations of carcinoid syndrome, such as flushing or secretory diarrhea, were not documented before presentation. In retrospect, the combination of isolated severe right-sided valvular disease with characteristic echocardiographic morphology should have prompted earlier consideration of CHD and evaluation for an underlying neuroendocrine tumor. 

Our patient presented with rapidly progressive right-sided heart failure leading to cardiogenic shock on admission, an uncommon presentation of CHD. This presentation has rarely been reported, particularly in the absence of an overt precipitating event such as carcinoid crisis, underscoring the need for clinicians to maintain a high index of suspicion in patients presenting with severe right-sided valvular disease. Typically, the disease develops slowly, reflecting progressive right-sided heart failure, such as exertional dyspnea, peripheral edema, and ascites, while many patients may have no noticeable symptoms in the early stages [1]. Less commonly, cardiac involvement may also manifest with arrhythmias, including atrial arrhythmias and ventricular tachycardia, likely related to the arrhythmogenic effects of circulating serotonin [8]. 

Although cardiogenic shock is an uncommon presentation of CHD, isolated cases have been reported. Published cases describe either progressive hemodynamic deterioration due to advanced carcinoid valvular disease or acute circulatory collapse associated with carcinoid crisis, highlighting distinct pathophysiological mechanisms and therapeutic approaches [9,10]. Our patient is more consistent with the former, given the extensive right-sided valvular fibrosis and severe valvular regurgitation without evidence of carcinoid crisis. Furthermore, recent surgical series have shown that advanced CHD remains associated with poor outcomes despite multidisciplinary management [11]. A summary of previously published cases and case series is provided in Table 1. Alternative endocrine causes of cardiogenic shock should also be considered in patients with atypical presentations. Acute adrenal insufficiency and adrenal crisis have been reported to cause severe myocardial dysfunction and cardiogenic shock; however, there was no clinical evidence suggesting adrenal crisis in our patient.

**Table 1:** Published reports of acute heart failure and cardiogenic shock in carcinoid heart disease. LV, left ventricular; NYHA, New York Heart Association

Study	Clinical entity	Mechanism	Clinical presentation	Management/Outcome
Present case	Advanced carcinoid heart disease	Progressive hemodynamic deterioration due to severe carcinoid valvular disease	Refractory cardiogenic shock with severe right-sided heart failure and multiorgan dysfunction	Medical therapy with lanreotide and supportive care; patient died before valve intervention
Ghukasyan et al. (2022) [9]	Advanced carcinoid heart disease (Hedinger syndrome)	Progressive serotonin-mediated fibrotic degeneration of right-sided valves	Acute decompensated right-sided heart failure, anasarca, flushing, and diarrhea	Progressed to cardiogenic shock requiring emergent valve replacement; died from postoperative mixed cardiogenic and distributive shock.
Maddali et al. (2021) [10]	Carcinoid crisis-induced acute systolic heart failure	Massive release of vasoactive substances causing transient myocardial dysfunction	Acute cardiogenic shock with new-onset severe LV systolic dysfunction	Mechanical circulatory support with subsequent recovery after treatment of carcinoid crisis
El Gabry et al. (2023) [11] (single-center surgical series)	Advanced carcinoid heart disease	Progressive right-sided valvular fibrosis requiring surgery	Eleven patients with NYHA III-IV heart failure undergoing valve surgery	30-day mortality of 18%; overall mortality of 72.7% during follow-up despite surgical intervention.

Prompt recognition of CHD is essential, since delays in treatment are linked to worse clinical outcomes and increased mortality [12]. 5-HIAA, a metabolite of serotonin, is one of the most useful tools in daily practice, but its interpretation may be influenced by dietary intake and pharmacological agents. Urinary 5-HIAA levels above 300 μmol/24 h have been associated with a 2-3-fold higher risk of cardiac involvement, and in serum, each 100 nmol/L increase has been related to a greater risk of disease progression [13]. NT-proBNP has the best overall diagnostic performance. A cutoff of 260 pg/mL provides a sensitivity of 92% and specificity of 91% for CHD [14]. In our patient, biomarkers were markedly elevated, a pattern that has been associated with worse prognosis and higher mortality [15].

Transthoracic echocardiography is the main imaging tool for the diagnosis of CHD and requires a structured evaluation of tricuspid and pulmonary valve involvement, right ventricular size and function, left-sided valves, the presence of a patent foramen ovale using agitated saline contrast, and possible cardiac metastases [16]. Typical findings of tricuspid valve disease are annular dilation and diffusely thickened, retracted leaflets with reduced mobility, resulting in incomplete systolic coaptation and limited diastolic opening, which explains the severity of regurgitation usually observed. The Westberg score, based on tricuspid valve anatomy and the degree of regurgitation, represents a simple and practical screening approach [17].

Current European Neuroendocrine Tumor Society recommendations support routine echocardiographic screening in patients with carcinoid syndrome, every year in those without cardiac involvement and every six months in patients with established disease. Cardiac magnetic resonance becomes particularly valuable when echocardiographic windows are suboptimal, as it allows more accurate assessment of the valves, quantification of regurgitant volumes, and contributes to surgical planning. Cardiac CT is mainly reserved for preoperative coronary evaluation and for the assessment of myocardial metastases [3].

The medical management of CHD is challenging, since both the underlying malignant condition and the cardiac involvement must be treated at the same time [7]. Long-acting somatostatin analogues remain the backbone of therapy for symptom control. Current treatment strategies aim to reduce urinary 5-HIAA levels to below 300 μmol/24 hour, although this recommendation is based on low-level evidence, and somatostatin analogues have not been shown to reverse established CHD [18]. In our patient, lanreotide was initiated as part of this strategy, given its established role in symptom control and disease stabilization in patients with neuroendocrine tumors and carcinoid syndrome [19].

However, in our patient, disease-directed therapy alone was unlikely to achieve hemodynamic recovery because of the advanced stage of cardiac involvement at presentation. Massive tricuspid regurgitation, marked right ventricular remodeling, and established refractory cardiogenic shock reflected irreversible structural cardiac disease. Although conventional echocardiographic indices of right ventricular systolic function were preserved or borderline, these parameters should be interpreted with caution in the setting of massive tricuspid regurgitation, as they may overestimate true right ventricular contractile performance because of altered loading conditions. At this advanced stage, somatostatin analogues may slow further disease progression but cannot reverse valvular fibrosis or restore normal right ventricular function. In cases with an inadequate response, treatment options include dose escalation of somatostatin analogues, addition of interferon alfa, or the use of peptide receptor radionuclide therapy [1].

In parallel, for patients who develop heart failure, therapy relies primarily on diuretic strategies, including loop and thiazide diuretics; other medications used in heart failure have not been studied in this condition. Evidence guiding the management of cardiogenic shock in CHD is limited, and no specific inotropic agent has been validated in this population. Inodilators such as levosimendan and milrinone are theoretically attractive because they augment right ventricular contractility while reducing pulmonary vascular resistance without relying on β-adrenergic stimulation. In our patient, levosimendan was selected and combined with norepinephrine for vasopressor support. She remained hemodynamically dependent on this therapy, with repeated failed weaning attempts over several weeks, reflecting the severity and instability of her underlying cardiac disease.

Surgical valve replacement is the only curative therapy for CHD, particularly for patients with severe valvular regurgitation and clinical symptoms. Surgical valve replacement improves symptoms, functional status, and quality of life and may confer a survival benefit; however, surgery carries a significant risk, with mortality ranging from 5% to 10% and increasing substantially in patients presenting with cardiogenic shock [20]. Bioprosthetic valves are preferred in most cases to avoid long-term anticoagulation, which is especially important in patients with advanced liver disease, metastatic burden, and frequent need for invasive procedures, as well as to reduce the thrombotic risk associated with mechanical valves in the right heart. For patients at very high surgical risk, percutaneous catheter-based interventions may be considered a minimally invasive alternative, although further studies are needed to clarify their role in the management of this condition.

Limitations

This report has important limitations. The absence of a reported Ki-67 proliferation index and WHO grade, together with the inability to identify the primary tumor site, limited definitive prognostic stratification and assessment of tumor-directed treatment options.

Learning points

CHD should be considered in patients with unexplained severe right-sided valvular disease, systemic congestion, and evidence of neuroendocrine malignancy. Characteristic echocardiographic findings, including diffuse right-sided valvular thickening, leaflet retraction, and severe tricuspid and pulmonary regurgitation, should prompt biochemical evaluation with urinary 5-HIAA and investigation for an underlying neuroendocrine tumor. Cardiogenic shock is an uncommon manifestation of CHD and usually reflects advanced right ventricular failure, emphasizing the importance of early recognition before definitive valve intervention is no longer feasible.

## Conclusions

We describe a patient in whom advanced carcinoid heart disease presented with refractory cardiogenic shock, highlighting that this rare presentation often reflects end-stage right-sided valvular disease. Although uncommon, this presentation should be recognized in patients with unexplained severe right-sided valvular dysfunction and systemic congestion, as timely diagnosis may allow disease-directed therapy and referral for valve intervention before irreversible hemodynamic deterioration occurs.
